# Gaston’s Operation

**Published:** 1885-01-20

**Authors:** 


### "GASTON'S OPERATION."

We hereby express our acknowledgements to Prof. J. McF. Gaston, of the Southern Medical College, Atlanta, for the reprint from Gaillard's Medical Journal, giving a somewhat extended notice of obstruction of the gall-ducts, with his suggestion of a new process for conveying the bile into the duodenum. It is accompanied with some editorial comments, stating that "Gaston has succeeded in his effort, and has created a new, bold, and life-saving operation." But we observe that some of our exchanges take issue with the designation of this as "Gaston's Operation" owing to the fact that Dr. Winiwerter had previously performed an operation for "attaching the gall-bladder to the colon"

If the physiological doctrine of the important role of the bile to the cholepoetic viscera be well founded (as we consider it is), there is a very wide difference in the result of carrying the bile into the duodenum and into the colon; so that there is really no ground for the claim of precedence in favor of Winiwerter's operation, as reported in the Prager Medezinische Wochenschrift, in 1882, over Gaston's operation having a totally diverse aim.

Dr. Gaston gives the result of "some experiments upon dogs, which are calculated to illustrate the practicability of uniting the walls of the gall-bladder and duodenum by an elastic ligature, and encircling this with stitches of catgut, so as to effect adhesion of the surrounding surfaces, when the opening is made by this constriction and cutting of the tissues ;" and states that all the stages of these successful experiments have been verified at different times by fifteen of his colleagues in this city.

A comparison of this process for utilizing the bile by delivering it into its proper channel, with others which remove it entirely from its appropriate sphere either by directing into the colon or turning it outside of the abdominal wall, as in cholecystotomy, must be favorable to the adoption of the remedial operation suggested by Professor Gaston. This appeal to the medical profession by the author is made without having had any opportunity to carry out his views upon the human subject; and, with the lights before them, surgeons must determine for themselves as to the practicability of undertaking this transformation of an abnormal into a normal condition by the restoration of the bile to the duodenum. In extreme cases, extreme measures are warranted ; and the junction of the walls of the gall-bladder with those of the duodenum, when the parts are already in a state of subacute inflammation, cannot add greatly to the dangers attending cholecystotomy. The fact is clearly established by the experiments on dogs that "a fistulous opening through the adjoining walls results from the action of even a simple silk ligature attaching the sac to the intestine, the bile being delivered" where it belongs, and of right ought to be, so that it only remains to test its practical application to man for this operation to be recognized among the approved surgical procedures of this progressive age.

